# Publisher’s Note

**DOI:** 10.1080/21655979.2024.2326372

**Published:** 2024-03-01

Due to a publisher error, a retraction notice was incorrectly issued on the article ‘Knockdown of long non-coding RNA AGAP2-AS1 suppresses the proliferation and metastasis of glioma by targeting microRNA-497-5p’, https://doi.org/10.1080/21655979.2021.1995573. We have now removed the retraction notice.

We apologise to the authors for this error.

